# Alteration of Brain Functional Networks in Early-Stage Parkinson’s Disease: A Resting-State fMRI Study

**DOI:** 10.1371/journal.pone.0141815

**Published:** 2015-10-30

**Authors:** Linqiong Sang, Jiuquan Zhang, Li Wang, Jingna Zhang, Ye Zhang, Pengyue Li, Jian Wang, Mingguo Qiu

**Affiliations:** 1 Department of Medical Imaging, College of Biomedical Engineering, Third Military Medical University, Chongqing, China; 2 Department of Radiology, Southwest Hospital, Third Military Medical University, Chongqing, China; Wake Forest School of Medicine, UNITED STATES

## Abstract

Although alterations of topological organization have previously been reported in the brain functional network of Parkinson’s disease (PD) patients, the topological properties of the brain network in early-stage PD patients who received antiparkinson treatment are largely unknown. This study sought to determine the topological characteristics of the large-scale functional network in early-stage PD patients. First, 26early-stage PD patients (Hoehn and Yahr stage:1-2) and 30 age-matched normal controls were scanned using resting-state functional MRI. Subsequently, graph theoretical analysis was employed to investigate the abnormal topological configuration of the brain network in early-stage PD patients. We found that both the PD patient and control groups showed small-world properties in their functional brain networks. However, compared with the controls, the early-stage PD patients exhibited abnormal global properties, characterized by lower global efficiency. Moreover, the modular structure and the hub distribution were markedly altered in early-stage PD patients. Furthermore, PD patients exhibited increased nodal centrality, primarily in the bilateral pallidum, the inferior parietal lobule, and the medial superior frontal gyrus, and decreased nodal centrality in the caudate nucleus, the supplementary motor areas, the precentral gyrus, and the middle frontal gyrus. There were significant negative correlations between the Unified Parkinson Disease Rating Scale motor scores and nodal centralities of superior parietal gyrus. These results suggest that the topological organization of the brain functional network was altered in early-stage PD patients who received antiparkinson treatment, and we speculated that the antiparkinson treatment may affect the efficiency of the brain network to effectively relieve clinical symptoms of PD.

## Introduction

Parkinson's disease (PD) is a degenerative disorder of the central nervous systemcharacterized by cardinal motor symptoms that results from the progressive degeneration of dopaminergic neurons in the nigrostriatal pathway. The pathological process underlying PD requires years to fully develop in the human nervous system, primarily in the brainstem, and the effects of PD reach the neocortex in its final stage[1]. Aside from its motor symptoms, nonmotor dysfunctions have been observed in PD[2–5]. Moreover, some early nonmotor symptoms have been detected during the preclinical stages of PD [6, 7], and postmortem studies indicate that pathologic processes may occur in mesolimbic regions during the presymptomatic phase of PD[1].Although the characteristic syndromes of PD are associated with dysfunction in the dopaminergic system, Dickson et al.[8]found that early clinical nonmotor features are more likely to be associated with pathological mechanisms aside from the dopaminergic system.

As a multi-system disease, some deficits in PD are suggested to arise from alterations in integrity of distributed brain neural networks[9].Resting-state functional magnetic resonance imaging (Rs-fMRI) is a non-invasive imaging technique used to investigate the integration of neural networks at resting state[10, 11]. Currently, many researchers have applied the Rs-fMRI technique to investigate the characteristics of the functional network in PD patients, and found impaired functional connectivity[12–14]. These studies mainly focused on functional connectivity, either within a whole-brain network or between different brain system. It is important to investigate the integrative brain network of functionally interacting brain regions.

In recent years, graph theory has been proved to be a powerful tool to character the global topological organization of brain networks[15–17], and has been applied to many psychiatric disorders and neurological diseases to detect pathologic mechanisms and biological markers; for instance, disrupted organization of the brain network has been observed in Alzheimer’s disease[18, 19], schizophrenia[20, 21]and major depressive disorders [22].

Graph theory has also been applied to investigate the abnormal functional brain network in PD patients. Furthermore, several studies have found an abnormal brain network in PD patients. For example, one study [23]found that global functional connectivity was reduced in the supplementary motor area (SMA), the dorsolateral prefrontal cortex and the putamen, and this reduction likely contributes to the motor impairment caused by PD. Based on wavelet correlation analysis, PD patients exhibited a marked decrease in nodal and global efficiency compared with healthy controls[24]. Baggio et al. [25] found that there are major reductions in long-range connections in PD patients with both mild cognitive impairment and no cognitive impairment. Recently, Luo et al. [26]found decreases in the local efficiency and local clustering coefficient in the weighted brain network of early-stage(Hoehn and Yahr stage: 1–2)drug-naive PD patients. Despite the increasing knowledge of topological organization of brain network of PD, however, previous studies mainly focused on mid-stage PD patients(Hoehn and Yahr stage: 2–3) who received antiparkinson treatment and early-stage drug-naive PD patients. According to European Federation of Neurological Societies, the levodopa therapy is the most effective therapy for early-stage PD patients to improve motor symptoms. Some previous studies also suggested that antiparkinson medication can partially restore the deficits in the functional brain network in PD patients [27, 28].The topological properties of large-scale functional brain network in early-stage PD patients who received antiparkinson treatment are poorly understood.

In this study, we sought to investigate the altered topological properties of the resting-state functional brain network in early-stage PD patients receiving antiparkinson treatment. Rs-fMRI data were collected from 26 early-stage PD patients(Hoehn and Yahr stage: 1–2) and 30 normal control subjects. Then, we reconstructed the functional brain network using nodes defined as 90 brain regions and edges defined as the temporal correlation coefficient between each region and the remainder of the brain. Subsequently, graph theoretical approaches were employed to analyze their topological properties, and between-group differences were found using a nonparametric test. Finally, the Pearson's correlation coefficient was computed to estimate the relationship between the functional network metrics and the Unified Parkinson Disease Rating Scale(UPDRS) motor scores.

## Methods and Subjects

### Subjects

A total of 26 right-handed early-stage PD patients (Hoehn and Yahr stage: 1–2) without dementia were consecutively recruited from Southwest Hospital (Table 1). All patients met the standard UK Brain Bank criteria for PD[29], and none had a history of head injury, stroke or other neurological diseases. The severity of motor symptoms on each side of the body was assessed by an experienced neurologist using the motor examination of the Unified Parkinson Disease Rating Scale (UPDRS),the Hoehn and Yahr disability scale [30], and the mini-mental state examination (MMSE) during off states. All patients had been unmedicated for at least 12 h before participation.

**Table 1:** Demographic characteristics and clinical status of each group.

	PD patients (n = 26)	Controls (n = 30)	P value
Age(years)	54.31± 10.90	56.81 ± 10.81	0.70^a^
Sex (male/female)	12/14	14/16	0.22^b^
Disease duration (years)	2.18 ± 1.52	NA	NA
UPDRS motor score (off medication)	18.96 ± 8.86	NA	NA
H&Y stage (off medication)	1.26±0.45	NA	NA
LEDD (mg/day)	317.96 ± 162.65	NA	NA
MMSE	28.4± 1.1	29.4 ± 1.2	0.36^a^

Abbreviations: PD, Parkinson's disease; UPDRS, Unified Parkinson's Disease Rating Scale; H&Y, Hoehn and Yahr; MMSE, mini-mental state examination; LEDD, levodopa equivalent daily dose; NA, not available.

^a^ The P value was obtained using the two-tailed two-sample *t*-test.

^b^ The P value was obtained using the chi-squared test. The data are presented as the means ± SD.

Additionally, 30 age- and sex-matched right-handed healthy controls participated in this study. All control subjects exhibited a normal neurological status and did not have a history of neurological diseases or psychiatric disorders. This study was approved by the local Medical Ethics Committee at Third Military University (Chongqing, China), and additional written informed consent was obtained from all patients for whom identifying information is included in this article.

### Data acquisition

All MRI data were obtained using a 3T scanner (Magnetom Trio; Siemens Medical Systems, Erlangen, Germany) equipped with eight-channel, phase-array head coils. Foam padding was used to minimize head motion by all subjects. For the resting-state scans, the subjects were instructed to remain still, relax and keep their eyes closed while not thinking of anything in particular. No subjects fell asleep according to a simple questionnaire after the scan. Functional images were acquired in the axial orientation using a single-shot, gradient-recalled echo planar imaging (EPI) sequence using the following parameters: TR = 2000ms,TE = 30 ms, flip angle = 90°, matrix size = 64×64, FOV = 192×192 mm^2^, 36 transverse slices,3-mm slice thickness without a gap, and resolution = 3×3×3 mm^3^.For each subject, a total of 240 volumes were acquired.

### Data processing

Data preprocessing was conducted using Statistical Parametric Mapping software (SPM8, http://www.fil.ion.ucl.ac.uk/spm) and Data Processing Assistant for Resting-State fMRI (DPARSF, http://www.restfmri.net). The first ten volumes were discarded to ensure steady-state longitudinal magnetization, and the remaining 230 volumes were corrected for the temporal difference in acquisition slice timing relative to the middle slice. The fluctuations in head motion were removed from the blood oxygen level-dependent signal using a multiple linear model containing six parameters (three translations and three rotations).For all subjects, the translational and rotational parameters of the data set did not exceed ±1.5 mm and±1°, respectively. Further, we used the two-tailed two-sample t test to estimate the differences in head motion between the PD group and the normal control group. There was no significant difference in head motion, which was measured astranslatory or rotatory movement, between the PD patients and the normal controls [translation: T = 0.87, p = 0.38;rotation: T = 1.29, p = 0.20]. Additionally, regression was performed to remove the signal from white matter and cerebrospinal fluid[31, 32].

Subsequently, the functional scans were spatially normalized to the standard Montreal Neurological Institute space using an optimum 6-parameter affine transformation and nonlinear deformations and were resampled to 3×3×3 mm^3^. To avoid introducing artificial local spatial correlations, no spatial smoothing was applied, as previously suggested[33, 34]. Finally, a band-pass filter (0.01–0.08 Hz) was used to reduce low-frequency drift and high-frequency physiological noise in each voxel [35].

In the functional brain network, the nodes represent brain regions. To determine the nodes of the functional brain network, an automated anatomical labeling (AAL) atlas [36] was employed to segment the brain into 90 regions of interest (ROIs) (45 for each hemisphere; S1 Table). The edges of the functional brain network represent the temporal correlation between each paired node. The time series was acquired for each ROI by averaging the signals of all voxels within that region[11, 37]. By calculating the Pearson correlation coefficient in the time series between each pair of ROIs, a 90×90 correlation matrix was obtained for each subject. Fisher’s r-to-z transformation was applied to the correlation matrix of each subject to improve the normality of the correlation coefficients [38]. Mean z-score absolute matrices for the normal controls and the PD patients are shown in S1 Fig. Finally, the individual correlation matrix of each subject was converted into a binary matrix (A_ij_ = [a_ij_]) according to a predefined threshold, where a_ij_ was 1 if the absolute correlation coefficient was higher than the threshold value and was 0 otherwise.

### Network analysis

#### Threshold selection

Here, we used the threshold sparsity, S, for all functional matrices. Using this approach, all resultant networks have the same number of nodes and edges, thereby enabling us to explore the between-group differences in the network organization. Each correlation matrix was repeatedly thresholded over a wide range of threshold levels rather than a single threshold. The sparsity range 0.1<S<0.35 with an interval of 0.01was selected for application to each correlation matrix. We chose the minimum threshold according to the criterion that the average degree (the degree of a node is the number of connections linked to the node) over all nodes of each thresholded network was larger than 2 * log(N) ≈9.5,whereN denotes the number of nodes, which was 90 in this analysis. The maximum threshold was obtained to ensure that each thresholded network displayed small-worldness across the sparsity range. Thus, we obtained unweighted binary graphs in which the nodes represented brain regions and the edges represented the temporal correlation between paired brain regions. Further network analysis was based on the 90×90 binary matrices for each subject across the entire sparsity range from 0.11 to 0.34, with an interval of 0.01.

#### Small-world properties

We employed a network efficiency measure to quantify the small-worldness of the functional brain network. This parameter can address the disconnected graphs dilemma and can provide a clear physical determinant of this topological property of the brain network.

The global efficiency E_glob_ is defined as follows [39]:

$$E_{glob}=\frac{1}{N\left(N-1\right)}\sum_{j\neq i\in G}\frac{1}{L_{ij}},$$

where L_ij_ is the shortest path length between nodes i and j. E_glob_ serves as a measure of parallel information transmission in the entire network [33].

The local efficiency of G is measured as follows [39]:

$$\;E_{loc}=\frac{1}{N}\sum_{i\in G}E_{glob}\left(i\right),$$

where E_glob_(i) is the global efficiency of G_i_. G_i_ is a subgraph that includes the nodes that connect to node i. Local efficiency measures the fault tolerance of the network, indicating the capacity for information exchange within each subgraph when the index node is eliminated [33].

Currently, the global efficiency of a small-world network is slightly less and the local efficiency is much greater than those of the matched random networks. Thus, the normalized global efficiency E_glob_(real)/E_glob_(random) ≈1, and the normalized local efficiency E_loc_(real)/E_loc_(random) >1. E_glob_(real) and E_loc_(real) represent the global efficiency and local efficiency in the real network, respectively. The global efficiency and the local efficiency of the random network were generated in accordance with the procedure described in a previous study[40, 41]. Using the Markov-chain algorithm, a random network was generated preserving the same number of nodes, number of edges and degree distribution as a real binary network for each sparsity. Then, we averaged across all 100 generated random networks to obtain a mean global efficiency E_glob_(random) and a mean local efficiency E_loc_(random)for each sparsity.

#### Network modularity

The modularity, Q, is a measure of the degree to which a network can be subdivided into well-delineated modules composed of densely interconnected nodes and few intermodular connections. To determine each subject’s community module structure, we used Newman’s spectral algorithm[42].

#### Nodal metrics

To examine the nodal characteristics of functional brain networks, we considered three nodal metrics: the nodal degree (K_i_), nodal efficiency (E_i_), and nodal betweenness centrality (B_i_).

K_i_ represents the number of connections to a node, which is defined as follows:

$$K_{i}=\sum_{i\neq j\in G}e_{ij},$$

where e_ij_ is the (i,j)th element in the binary correlation matrix.

E_i_ is defined as follows [33]:

$$E_{i}=\frac{1}{N-1}\sum_{i\neq j\in G}\frac{1}{L_{ij}},$$

where L_ij_ is the shortest path length between nodes i and j.

B_i_ is defined as follows [43]:

$$B_{i}=\sum_{i\neq j\neq k\in G}\frac{\delta_{jk}\left(i\right)}{\delta_{jk}},$$

Where *δ*_*jk*_ is the number of shortest paths from node j to node k and *δ*_*jk*_(*i*) is the number of shortest paths from node j to node k that pass through node i within graph G. B_i_ measures the quantity of information transmitted via node i between the rest node and the entire network [44]. Regions displaying high relative B_i_ were considered to be hubs in the brain network [45].

### Statistical analysis

#### Differences in network metrics

Graph metrics of the functional brain networks (E_glob_, E_loc_, K_i_, E_i_, and B_i_) were compared to determine whether there are significant differences between early-stage PD patients and controls. Specifically, we calculated the area under the curve (AUC) for each network metric to provide a summarized scalar for the topological characterization of brain networks independent of single threshold selection. The AUC of each metric Y was computed as follows:

$$Y^{AUC}=\sum_{k=1}^{n-1}[Y(S_{k})+Y(S_{\left(k+1\right)})]\Delta S/2.$$

In this study, S_1_ = 0.11, S_n_ = 0.34, and Δ*S* = 0.01. The AUC of metrics, which has been used in several previous studies, can detect alterations in some diseases [22, 46].

Subsequently, the nonparametric permutation test was performed on the AUC of each network metric to assess between-group differences. First, we calculated the between-group difference of each graph metric of the functional brain networks. Next, each participant was randomly assigned to one of two groups of the same size as the original groups of patients and healthy controls. This randomization procedure was repeated for 10000 permutations, generating a null permutation distribution. The new between-group difference was calculated for each permutation. Then, we assigned a p value for each between-group difference by computing the proportion of differences exceeding the null distribution values. A p value of less than 0.05 was considered to indicate statistical significance. Notably, before the permutation test, the effects of confounding factors, including age and gender, were removed via multiple linear regression.

#### Relationships between network measures and clinical variables

Once significant between-group differences in any network metric were observed, we further assessed the relationships between these metrics and the UPDRS motor scores by computing the Pearson's correlation coefficient (p<0.05).

#### Choice of hub regions across the threshold range

The B_i_ values are threshold-dependent; thus, these values could vary from one threshold to another. To quantify this variability, we applied a nonparametric one-tailed sign test. For each brain region, this test was performed considering the null hypothesis that the given brain region was not a hub, that is, $B_{i}^{total}\leq mean\left(B_{net}^{total}\right)+SD\left(B_{net}^{total}\right)$, and corrections for multiple comparisons were performed (p<0.002,FDR-corrected).

## Results

### Global network topological properties

**Fig 1:**
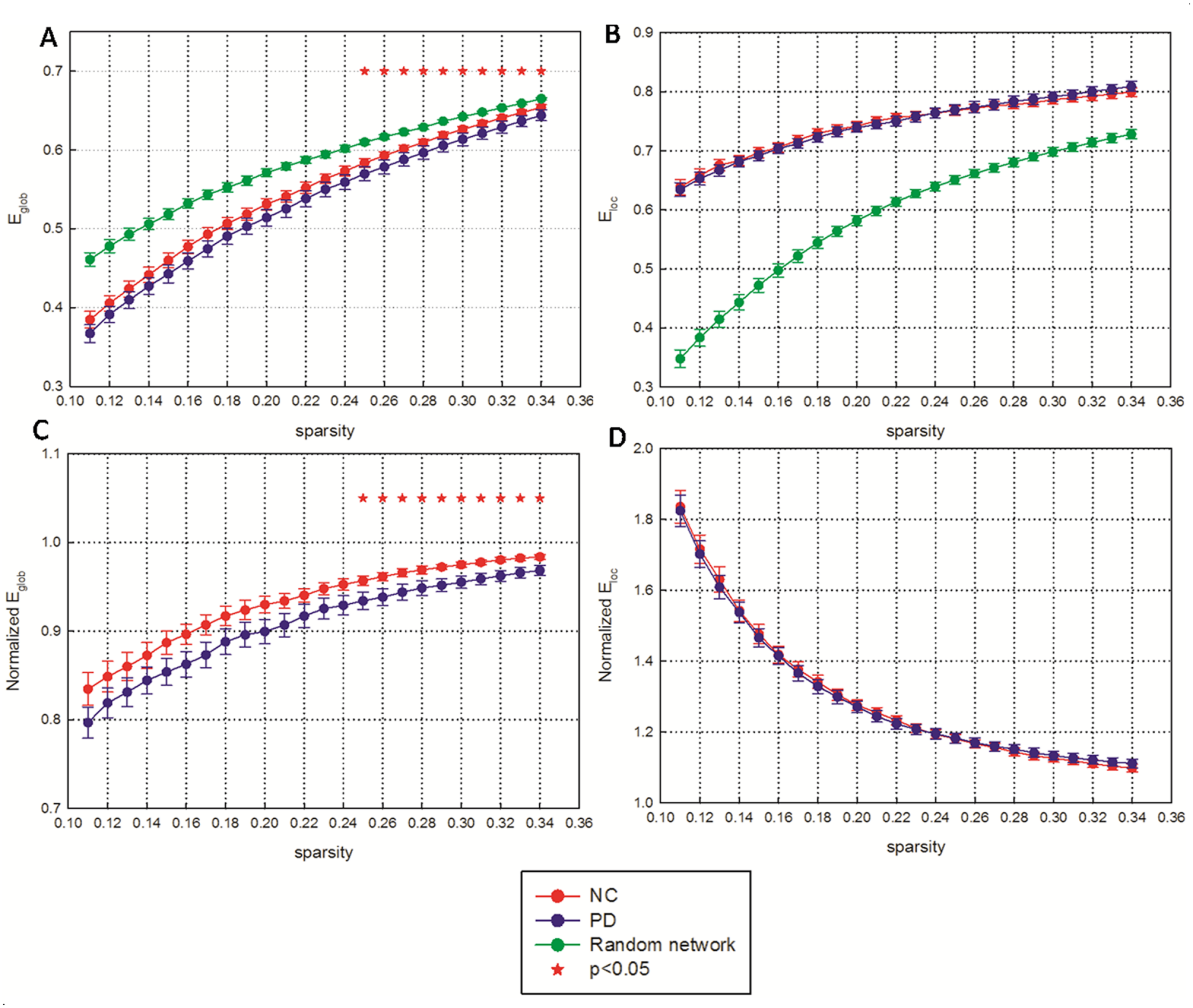
Network efficiency of functional brain networks as a function of sparsity among early-stage PD patients and normal controls. (A) Mean global efficiency and (B) mean local efficiency of the functional brain networks. (C) Normalized global efficiency and (D) normalized local efficiency of the functional brain networks. The functional brain networks showed increased local network efficiency but approximately identical global network efficiency of parallel information transmission compared with the matched random network. Error bars denote standard deviations. Red stars indicate statistically significant differences between the PD patients and the normal controls (p < 0.05). NC: normal controls; PD: Parkinson's disease.

**Fig 2:**
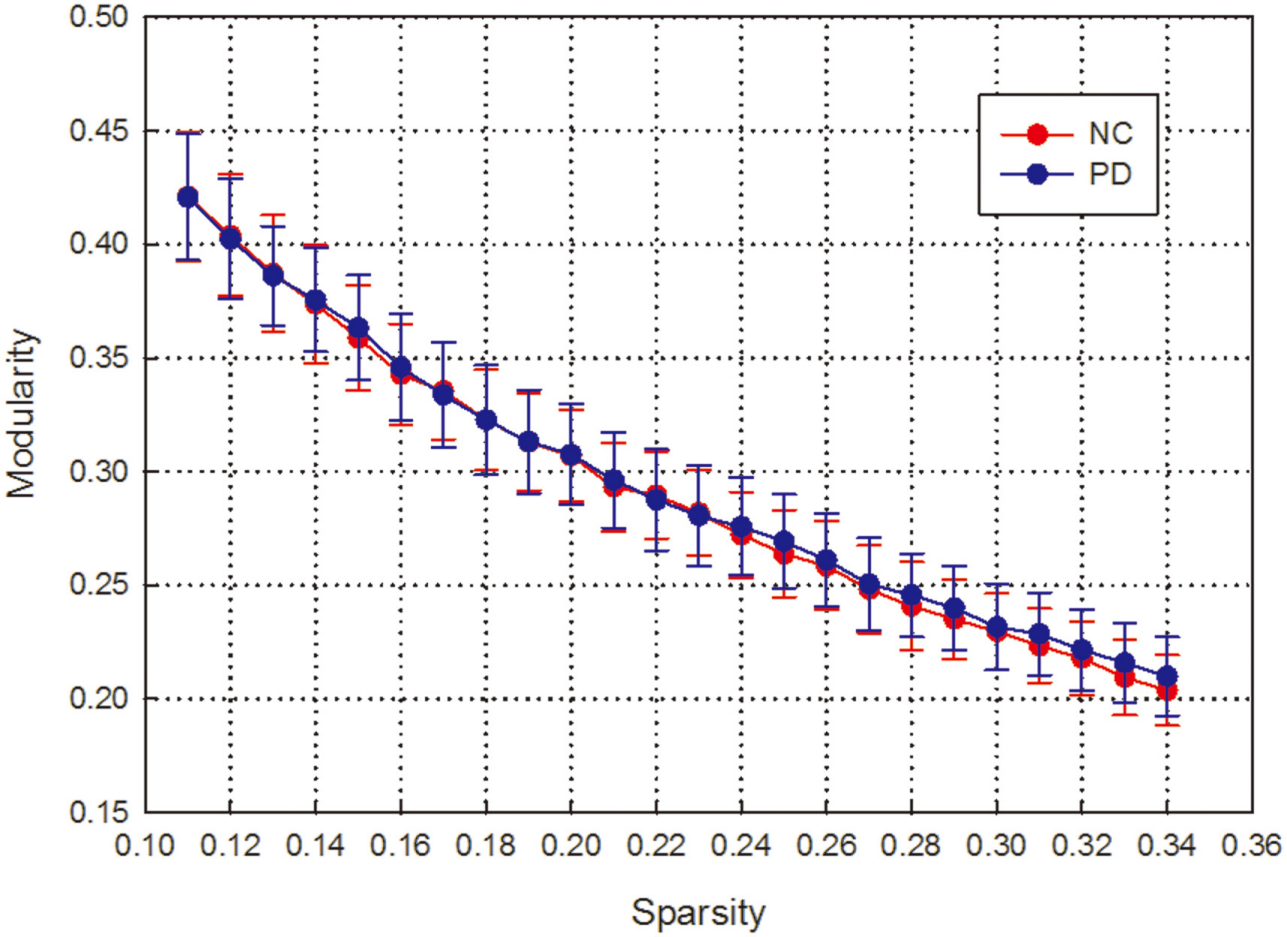
Network modularity of functional brain networks as a function of sparsity among PD patients and normal controls.

We observed that the normalized local efficiency is much greater and the normalized global efficiency is slightly less than 1 in a wide range of sparsity in both healthy controls and early-stage PD patients. This result indicates that the local efficiency is much higher and the global efficiency is slightly lower in the functional networks of these cohorts than in the matched random networks (Fig 1). Modularity was also observed in the two groups at a wide sparsity range (Fig 2). Moreover, we found that both the global efficiency and the normalized global efficiency were lower in PD patients than in healthy controls, and these differences were significant at relatively high thresholds of sparsity(two-tailed two-sample t-test, p<0.05). In contrast, there was no significant difference in local efficiency, normalized local efficiency or modularity between the two groups at any sparsity threshold.

Furthermore, the nonparametric permutation test performed on the AUCs of each network metric revealed that the PD patients exhibited significantly lower global efficiency (p = 0.04) and normalized global efficiency (p = 0.04) than the healthy control subjects. No significant difference in local efficiency, normalized local efficiency or modularity of the functional brain networks was observed between the PD patients and the control subjects (p>0.05).

### Modularity of the functional brain network

**Fig 3:**
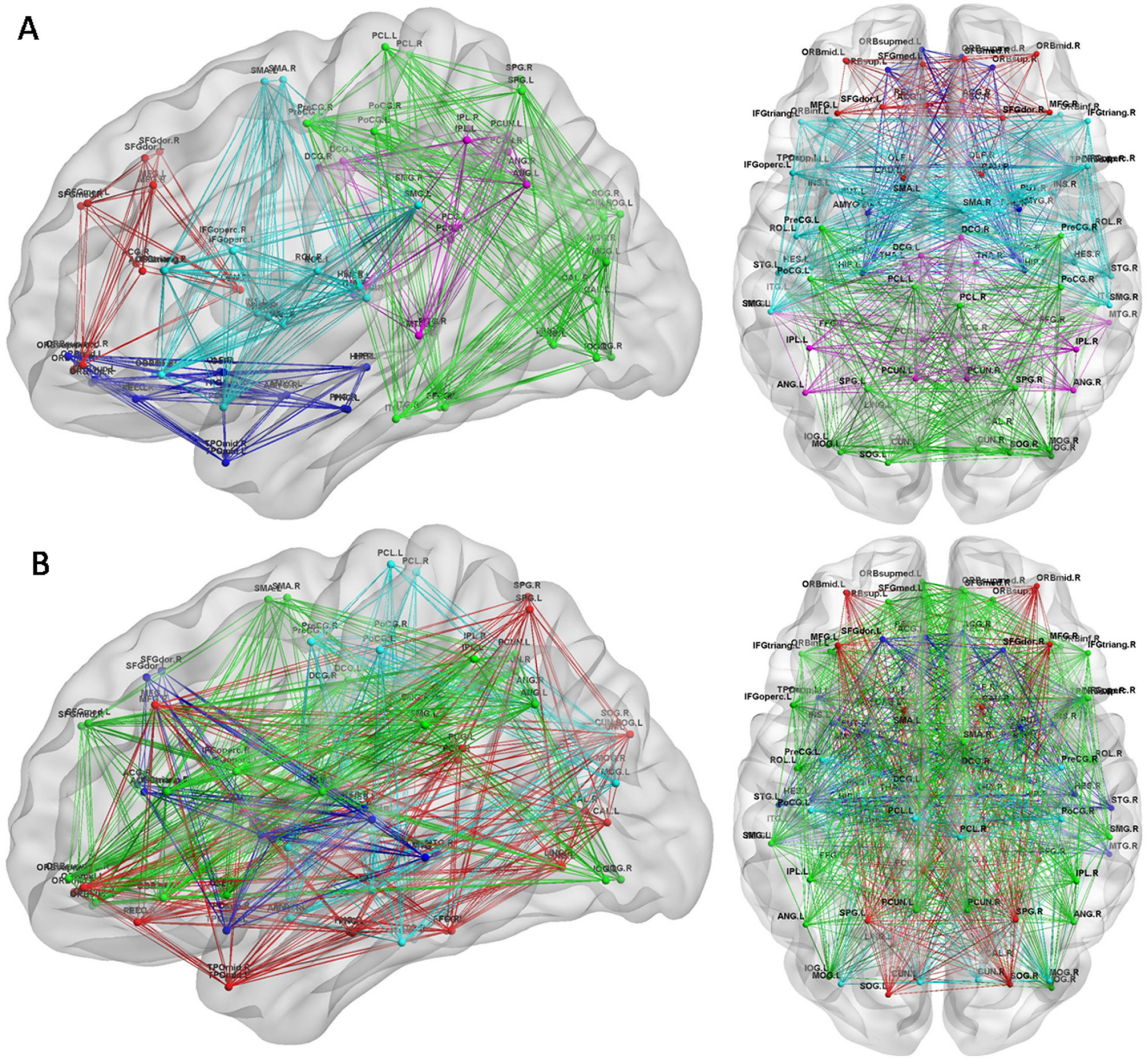
Modules of the functional brain networks in each group. (A) Modules of the brain network in normal controls. (B) Modules of the brain network in PD patients. Colors in nodes and links correspond to different modules. Only intramodular edges are shown. The results were visualized using the BrainNet Viewer (NKLCNL, Beijing Normal University).

The mean brain networks of the PD patient group and the control group were decomposed into four and five modules, respectively, using Newman's modularity algorithm (Fig 3). An additional module composed of the thalamus, the posterior cingulate gyrus, the inferior parietal lobule, the angular gyrus and the middle temporal gyrus was identified in the normal controls; most of these brain regions are components of a default mode network. The mean number (SD) of modules was 2.88(0.9) for the PD patients and 3(0.64)for the controls. No significantly different group effect was found for the number of modules (two-tailed two-sample t-test, p = 0.5;T = 0.55) in early-stage PD patients.

### Network hub distribution

**Fig 4:**
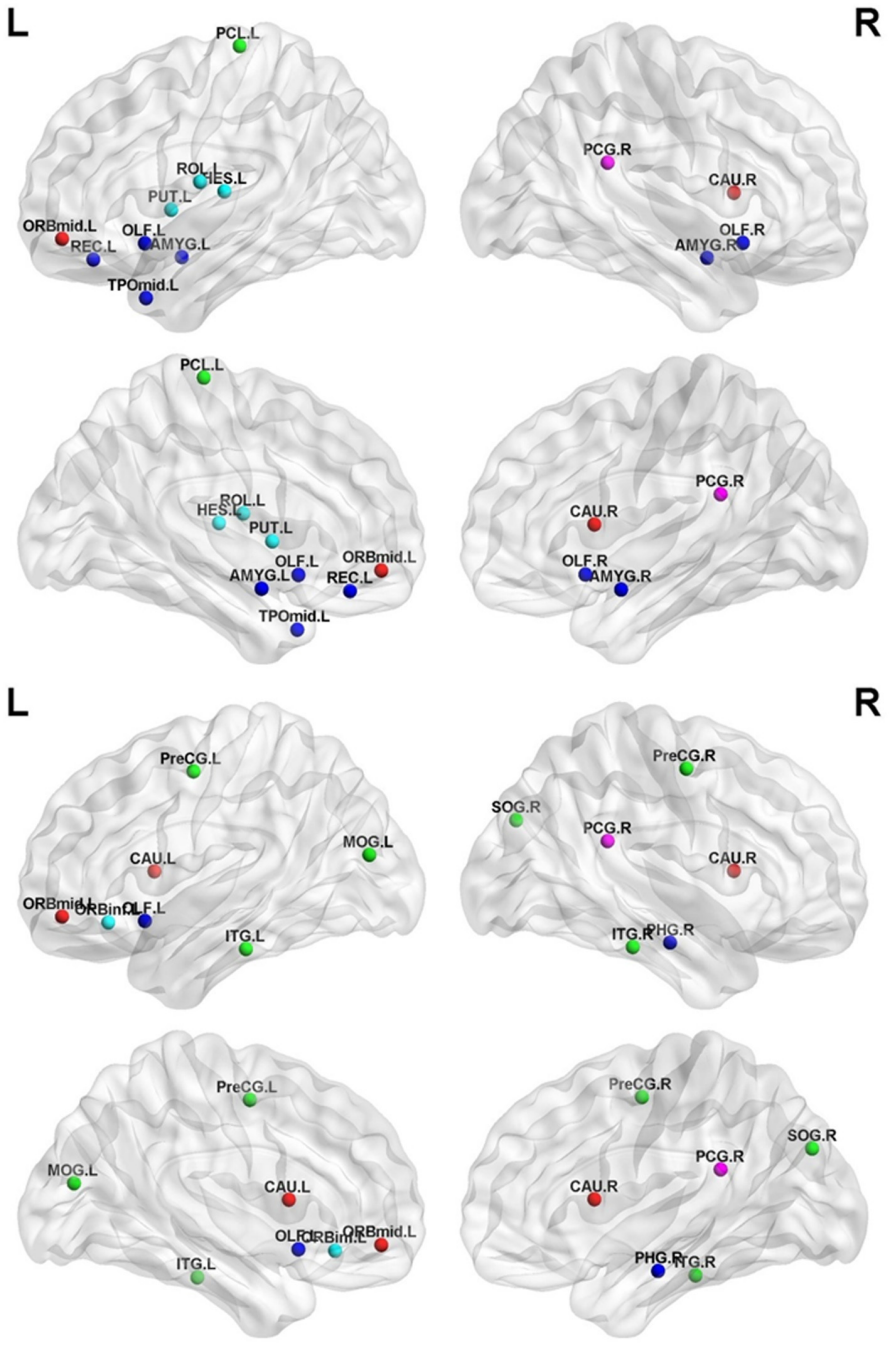
Hubs of the functional brain networks in each group. The results were visualized using the BrainNet Viewer (NKLCNL, Beijing Normal University). The maps showed the hubs of the brain network in normal controls (top panel) and in PD patients (bottom panel). Colors in nodes indicate different modules. Detailed brain region information corresponding to the anatomical labels can be found in S1 Table.

The highly reproducible and consistent hubs based on the betweenness centrality across all threshold levels are shown in Fig 4. There were13 highly reproducible hubs in both PD patients and normal controls (p<0.002, FDR-corrected). The hubs in the normal controls were the bilateral olfactory cortex and amygdala, the left middle frontal cortex(orbital part), rolandic operculum, rectus, paracentral lobule, putamen, Heschl’sgyrus, and middle temporal poleas well as the right posterior cingulate gyrusand caudate (Fig 4A). The hubs in PD patients were the bilateral precentral gyrus, caudate, and inferior temporal gyrus; the left middle frontal gyrus (orbital part), inferior frontal gyrus (orbital part), olfactory bulb, and middle occipital gyrus; and the right posterior cingulate gyrus, parahippocampal gyrus, and superior occipital gyrus (Fig 4B).

### PD-related alterations in regional nodal centrality

**Fig 5:**
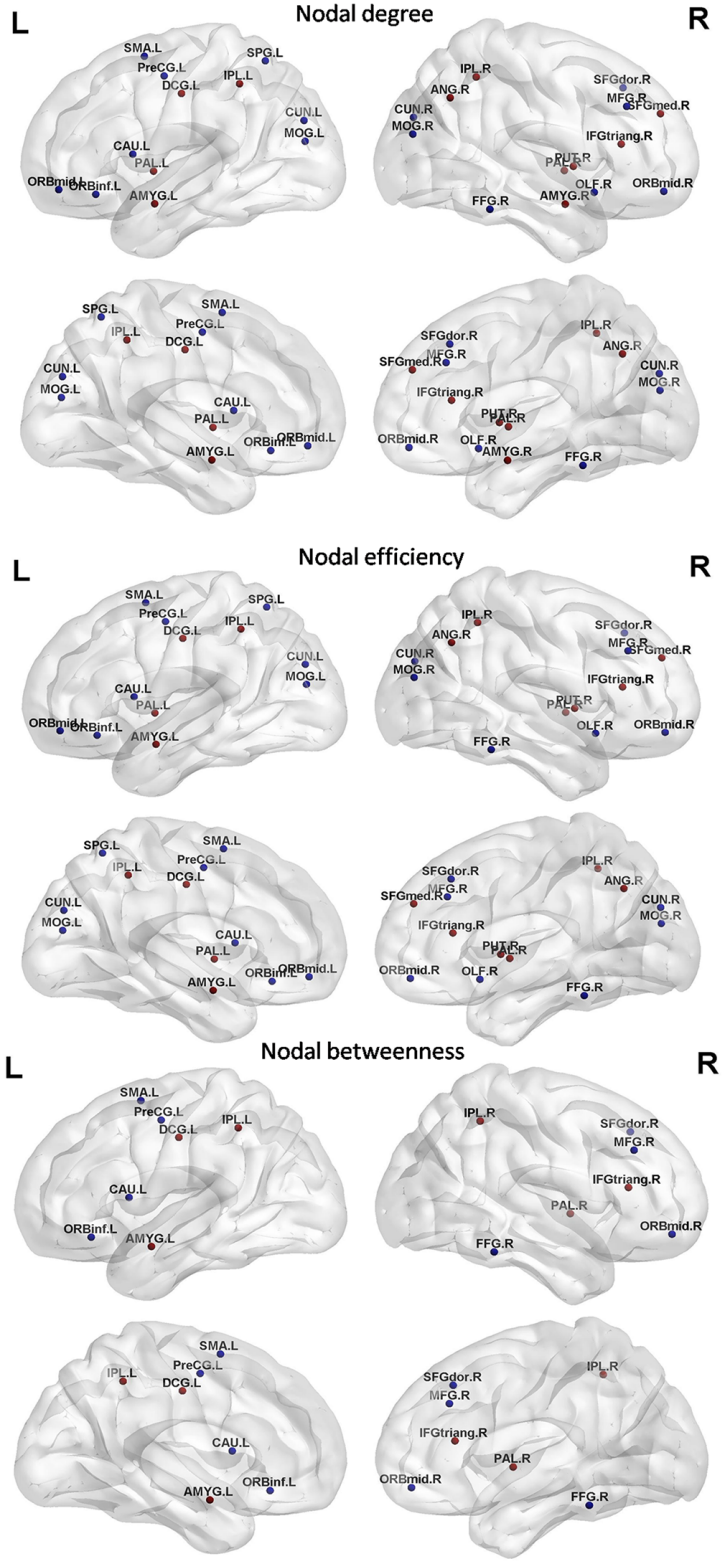
Brain regions showing significant alterations in nodal centrality between PD patients and normal controls (p<0.05, uncorrected). The results were visualized using the BrainNet Viewer (NKLCNL, Beijing Normal University).Three-dimensional maps show the differences in nodal degree (top panel),nodal efficiency (middle panel),and nodal betweenness (bottom panel)between the PD group and the control group. Red/blue spheres denote regions of increased/decreased nodal centrality in PD patients. Detailed brain region information corresponding to the anatomical labels can be found in S1 Table.

We identified the brain regions showing significant between-group differences in at least one nodal metric (p<0.05,FDR-corrected) (Fig 5 and Table 2). Compared with healthy control subjects, PD patients showed increased nodal centrality in several brain regions, including the bilateral pallidum, the bilateral amygdala, the right medial superior frontal gyrus, and certain regions located in the parietal lobule. Decreased nodal centrality in PD patients compared with healthy controls was observed in several other regions, including the left SMA, caudate nucleus, and precentral gyrus; the right frontal gyrus and fusiform gyrus; and certain regions located in the occipital gyrus.

**Table 2:** Regions showing abnormal nodal centrality in PD patients compared with control subjects (p<0.05, FDR-corrected, shown in bold font).

Brain region	p values		
	Nodal degree	Nodal efficiency	Nodal betweenness
**PD patients>healthy controls**			
Right inferior frontal gyrus, triangular part	**<0.001**	**<0.001**	**0.006**
Right superior frontal gyrus, medial part	**0.012**	**0.007**	>0.05
Left medial cingulate and paracingulate gyri	**0.001**	**0.009**	>0.05
Left amygdala nucleus	**0.007**	**0.023**	>0.05
Right amygdala nucleus	**0.006**	>0.05	**0.023**
Left inferior parietal lobule	**0.014**	>0.05	**0.041**
Right inferior parietal lobule	**0.005**	**0.015**	**0.046**
Right angular gyrus	**<0.001**	**0.03**	>0.05
Left pallidum	**<0.001**	**0.002**	>0.05
Right pallidum	**<0.001**	**<0.001**	**0.005**
Right putamen	**0.004**	**0.005**	>0.05
**PD patients<healthy controls**			
Left precentral gyrus	**<0.001**	**<0.001**	**0.002**
Right dorsal lateral superior prefrontal gyrus	**0.042**	**0.021**	**0.015**
Right middle frontal gyrus	**0.006**	**<0.001**	**0.002**
Right middle frontal gyrus, orbital part	**<0.001**	**<0.001**	>0.05
Left middle frontal gyrus, orbital part	**<0.001**	**<0.001**	>0.05
Left inferior frontal gyrus, orbital part	**0.007**	**0.003**	**0.001**
Left supplementary motor area	**0.018**	**0.005**	**0.024**
Right olfactory cortex	**0.004**	>0.05	>0.05
Left middle occipital gyrus	**0.001**	**0.01**	>0.05
Right middle occipital gyrus	**0.01**	>0.05	>0.05
Right fusiform gyrus	**<0.001**	**0.003**	**0.049**
Left cuneus	**0.002**	**0.002**	>0.05
Right cuneus	**<0.001**	**<0.001**	>0.05
Left superior parietal gyrus	**<0.001**	**<0.001**	>0.05
Left caudate nucleus	**<0.001**	**0.01**	**<0.001**

### Relationship between the network parameters and the clinical measures

**Fig 6:**
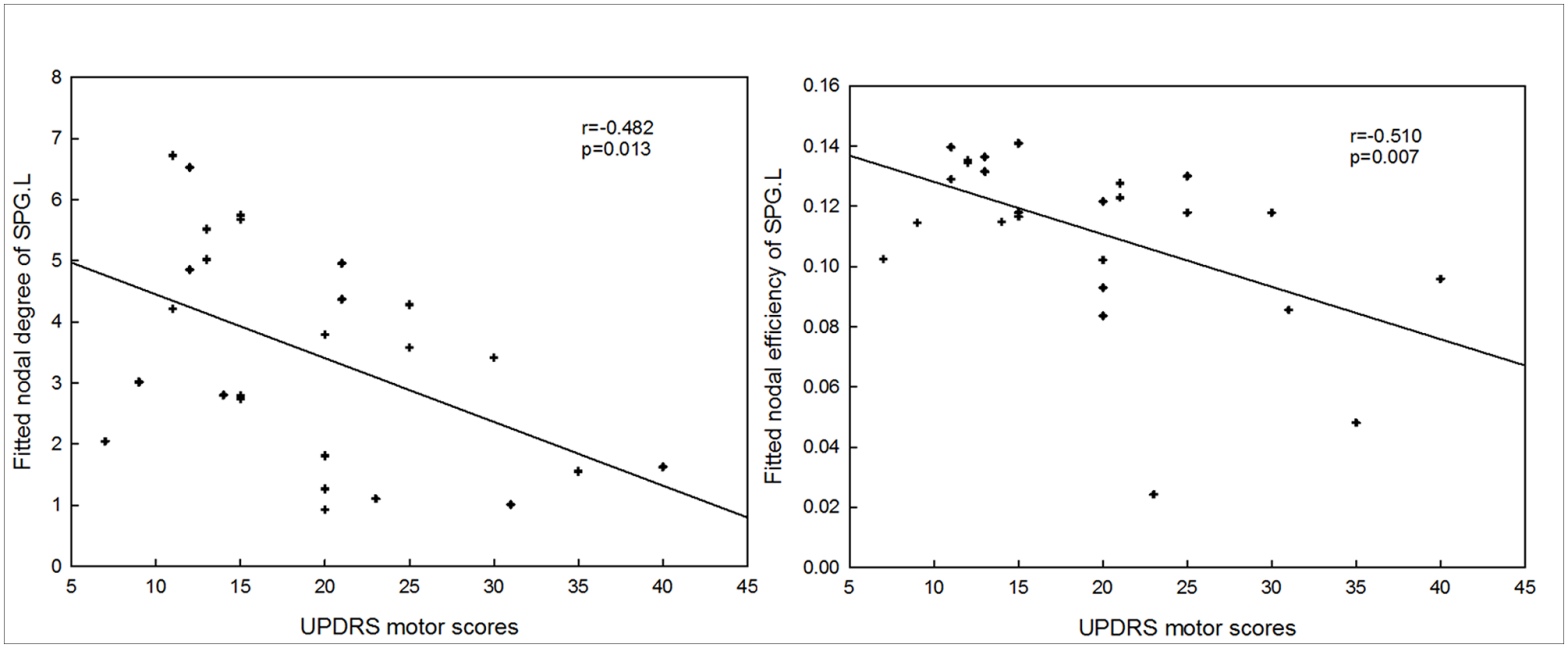
Correlation between abnormal network properties and UPDRS motor scores. SPG.L: the left superior parietal gyrus.

There was no significant correlation between the UPDRS motor scores and the global network metrics: global efficiency and normalized global efficiency. There were significantly negative correlations between UPDRS motor scores and nodal centralities of the left superior parietal gyrus (nodal degree: r = -0.482,p = 0.013; nodal efficiency: r = -0.510,p = 0.007) (Fig 6).

## Discussion

The present study investigated the topological properties of functional brain networks in early-stage PD patients who received antiparkinson treatment. The results revealed that global efficiency was decreased in early-stage PD patients, implying that the organization of the entire brain was disturbed. However, no significant difference in local efficiency was found between early-stage PD patients and normal controls. Moreover, the modularity and hub distribution were disrupted in early-stage PD patients. In addition, the nodal characteristics of several motor-related and cognitive-related brain regions were substantially altered in the early-stage PD patients. Thus, our results have provided more insights into understanding of topological mechanism of early-stage PD who received antiparkinson treatment.

### Altered network organization

Many previous studies have revealed that the human brain network displays small-worldness, which is characterized by an increased clustering coefficient and almost identical characteristic path lengths compared with a random network[33]. Recently, the small-world network was reported to be economical, with a low sparsity and high efficiency in propagating information [34, 39, 47]. In this study, we found that the functional networks of both PD patients and normal controls displayed small-worldness based on measures of efficiency (Fig 1).

Despite the common small-world topology demonstrated in the functional networks of both early-stage PD patients and normal controls, there were significant differences in theirs mall-world properties. The early-stage PD patients showed a significant decrease in global efficiency but no significant difference in local efficiency of the brain network compared with the controls (Fig 1). These results revealed that the balance between local segregation and global integration was disturbed in the functional brain network of PD patients. Significantly decreased global efficiency was associated with decreased long-range connections across remote cortical regions in PD patients (S2 Table). This decreased global efficiency indicated a reduced capacity of information transfer across the entire brain. Notably, several previous studies have found decreased global efficiency or long-range functional connectivity in mid-stage PD patients who had been chronically exposed to a large dose of antiparkinson medication. Skidmore et al.[24]found a marked decrease in global efficiency in PD patients compared with healthy controls based on wavelet correlation analysis. Baggio et al.[25]found widespread long-range connectivity decreases in a PD cohort, most notably in patients with mild cognitive impairment. Additionally, Dubbelink et al.[48] found reductions in global efficiency in association with PD progression based on magnetoencephalography. However, Luo et al.[26] found decreases in the local efficiency and local clustering coefficient in early-stage drug-naive PD patients. These previous results suggested that mid-stage PD groups who had received antiparkinson treatment showed decreased global efficiency and no significant difference in local efficiency, but early-stage drug-naive PD patients showed decreased local efficiency and no significant difference in global efficiency. Specifically, we found decreased global efficiency in early-stage PD group who received antiparkinson treatment, but almost identical local efficiency compared with normal controls (S3 Table).According to previous studies and our results, we speculated that the antiparkinson treatment may affect the efficiency of brain network to effectively relieve clinical symptoms.

### Altered modularity of brain network

Modularity is an important organizational principle of complex biological networks. Thus, investigating modularity might be very helpful in elucidating the topological properties of brain networks[17].Although there was no significant difference in modularity across the range of sparsity, the modular structure identified in early-stage PD patients was markedly altered(Fig 3). The most notable alteration was the detection of an additional modular structure in normal controls; this module contained the thalamus and several regions that are components of a default mode network. Moreover, no significantly different group effect was found for the number of modules (p = 0.5; T = 0.55) in early-stage PD group.

### Altered network hub distribution

We also found that early-stage PD was associated with a reorganization of the hub structure, characterized by the betweenness centrality (Fig 4). The hubs that were most notably altered in early-stage PD group were located in limbic system areas such as the parahippocampal gyrus and the amygdala, which regulates emotions and behavior and which may be associated with the degenerative pathogenic process of PD. The notable eliminated hub in the early-stage PD group compared with the normal control group was the putamen, which plays an important role in regulating movements; this alteration may contribute to the motor impairments observed in early-stage PD patients. In particular, altered nodal centrality was primarily localized to the hubs of both normal controls and PD patients (Table 2 and Fig 5); this alteration corresponded to the changes in the functional organization of the brain network in PD patients.

### Altered nodal centrality

In this study, several regions displaying abnormal nodal centrality are components of the basal ganglia. For instance, decreased centrality was observed in the left caudate nucleus, and increased nodal centrality was observed in the right putamen and the bilateral pallidum (Table 2 and Fig 5). In PD, the prominent degeneration of dopaminergic neurons of the substantia nigra pars compacta leads to aconsequent deficiency indopamine in basal ganglia regions; this deficiency triggers many functional changes affecting the entire basal ganglia network in PD [12].The basal ganglia circuit is functionally interposed between the cortex and the thalamus to modulate movement execution. Experimental studies show that the basal ganglia exerts an inhibitory influence on several motor systems and that releasing this inhibition permits the activation of a motor system. Several previous studies have found that the functional changes in the basal ganglia circuit are associated with PD[12, 23, 49]. Our results were in line with these previous studies, indicating that the basal ganglia circuit is disrupted in a relatively early stage of PD.

Additionally, the caudate nucleus in the basal ganglia is highly innervated by dopaminergic neurons and significantly contributes to body and limb posture as well as to the speed and accuracy of directed movements. Significant volumetric atrophy in the caudate nucleus was detected in a previous study[50]. The decreased nodal centrality in the caudate nucleus observed in the present study is compatible with the results of previous studies. Moreover, increased nodal centrality was detected in the pallidum. Within the basal ganglia, the pallidum integrates the inhibitory input from the striatum with the excitatory input from various regions—for instance, the cortex and the thalamus. These opposing inputs influence the output of the basal ganglia [51].The pallidum is subdivided into two components: the external pallidal segments(GPe) and the internal pallidal segments (GPi). A deficit indopamine in PD leads to excessive activation of the indirect pathway between the striatum and the pallidum[52], ultimately resulting in the disinhibition of the GPi/substantia nigra pars reticulata (SNr) output nuclei of the basal ganglia—i.e., increased neuronal activity in the GPi/SNr—leading to excessive inhibition of thalamo-cortical and brainstem motor systems, thereby producing the motor disorders associated with PD[53]. Increased nodal centrality in the pallidum suggests its strengthened role in the entire brain, presumably to compensatefor the motor disorders resulting from PD.

Moreover, a PD-related decrease in nodal centrality was observed in the left SMA and precentral gyrus and the right dorsal lateral superior prefrontal gyrus and superior parietal lobule, which are involved in motor functions (Table 2 and Fig 3). According to the model proposed by Alexander et al. [54], the major factor responsible for motor impairment is a deficiency in the activation of cortical areas involved in the motor loop, notably the SMA, which is considered as a key structure in the cortico-basal ganglia motor loop[55, 56]. Importantly, there were significantly negative correlations between UPDRS motor scores and nodal centralities of the left superior parietal gyrus (Fig 6), indicating that the higher UPDRS motor scores, the less important the role of the left superior parietal gyrus in information transmission within the brain network. Decreased nodal centrality in these motor areas indicates their reduced roles in the brain network, likely in response to the degenerative changes in motor function in early-stage PD. We also found a PD-related increase in nodal centrality in the inferior parietal lobule, which plays a key role in conscious intentional processes—e.g., the "wanting to move" intention [57].Increased nodal centrality in the inferior parietal lobule may compensate for the impairment of movement functions in PD patients. Taken together, these brain regions displaying abnormal nodal centrality are associated with motor management and motor executive functions, and these results confirm that abnormal nodal centrality in motor-related regions contributes to the motor impairment observed in PD.

In this study, we also found abnormal nodal centrality in several cognition-related brain regions (Table 2 and Fig 3).

Several previous studies have verified cognitive dysfunctions aside from the remarkable motor symptoms of PD[2–5]. Notably, it has been confirmed that certain early nonmotor symptoms appear during the preclinical stages of PD [6, 7].

There is mounting evidence that the basal ganglia plays important roles in cognitivefunction[58, 59].In particular, accumulating studies have linked the cognitive manifestations of PD—both early-stage and mid-stage PD—to dopaminergic dysfunction in the caudate nucleus [60–62].Presumably, altered nodal centrality of the basal ganglia contributes to the cognitive impairment observed in early-stage PD patients. Several regions of abnormal nodal centrality that are primarily located in the prefrontal cortex may also be associated with cognitive impairment in PD patients. However, there was no significant difference in the MMSE scores between the PD group and the normal control group (Table 1).

### Study limitation and future direction

Several limitations of this study should be addressed. First, the PD patients recruited for this study exhibited varying clinical symptoms, such as akinetic, tremor and mixed symptoms. Several previous fMRI studies [13] have indicated that different PD symptoms could result from distinct neuronal mechanisms—in other words, different PD symptoms correspond to distinct small-world characteristics in the functional brain network. In the future, we should stratify PD patients according to their symptoms and investigate whether these subgroups show distinct topological architectures in their brain networks. Second, the functional brain network was constructed at a regional level based on a previously published atlas (AAL) by parcellating the entire brain into 90 regions. Some regions verified to be related to PD, such as the substantianigra and the subthalamic nucleus, were not subdivided in the atlas used in this study. Brain networks constructed using different spatial scales show distinct functional organization. Future studies are needed to select different ROIs or different brain parcellation strategies to identify the most appropriate topological architectures for the brain networks of PD patients. Third, further experiment should be carry out to quantify the differences between early-stage PD who received antiparkinson treatment and early-stage drug-naive PD groups to evaluate the effect of antiparkinson treatment on topological properties of brain network. Finally, no detailed cognitive scale was used to verify the relationship between the abnormal nodal centrality of cognition-related brain regions and the cognitive impairment symptoms observed in PD patients in this study. In future, we will collect detailed cognitive scale to evaluated the cognitive impairment by PD.

## Conclusions

In this study, we focused on investigating the topological architectures of the functional brain networks in early-stage PD patients who received antiparkinson treatment. Compared with normal controls, early-stage PD patients exhibited a significant decrease in global efficiency but no significant difference in local efficiency. Alterations in modular structure and hub distribution were also observed in early-stage PD patients. Furthermore, altered nodal centrality was detected in several regions that are components of the basal ganglia, such as the caudate nucleus, the pallidum, and the putamen. We also found abnormal nodal centrality in certain motor-related regions, such as the SMA, and certain cognition-related regions, such as the prefrontal cortex. There was no significant correlation between the UPDRS motor scores and global network metrics, but significantly negative correlations were found between UPDRS motor scores and nodal centralities of the left superior parietal gyrus. In summary, these results suggest that the topological organization of the brain functional network altered in early-stage PD patients who received antiparkinson treatment, and provided more insights into understanding of topological mechanism of early-stage PD who received antiparkinson treatment. Additionally, according to previous studies and our results, we speculated that the antiparkinson treatment may affect network efficiency of brain network to effectively relieve clinical symptoms.

## Supporting Information

S1 Fig Mean z-score absolute matrices in both normal controls (NC) and PD patients groups. The x and y axes correspond to the ROIs listed in S1 Table. The functional connectivity is indicated with a colorbar.
(TIF)

S1 Table Anatomical regions of interest (ROIs). (DOCX)

S2 Table Decreased functional connectivity in PD patients compared with normal controls (p<0.002, uncorrected). (DOCX)

S3 Table The mean values of global efficiency and local efficiency in both normal controls and PD patients groups. (DOCX)
