# Supplementary figures and images for: Alteration of Brain Functional Networks in Early-Stage Parkinson’s Disease: A Resting-State fMRI Study
Source: PLoS One. 2015 Oct 30;10(10):e0141815. doi: 10.1371/journal.pone.0141815 (PMC4627652; doi:10.1371/journal.pone.0141815)

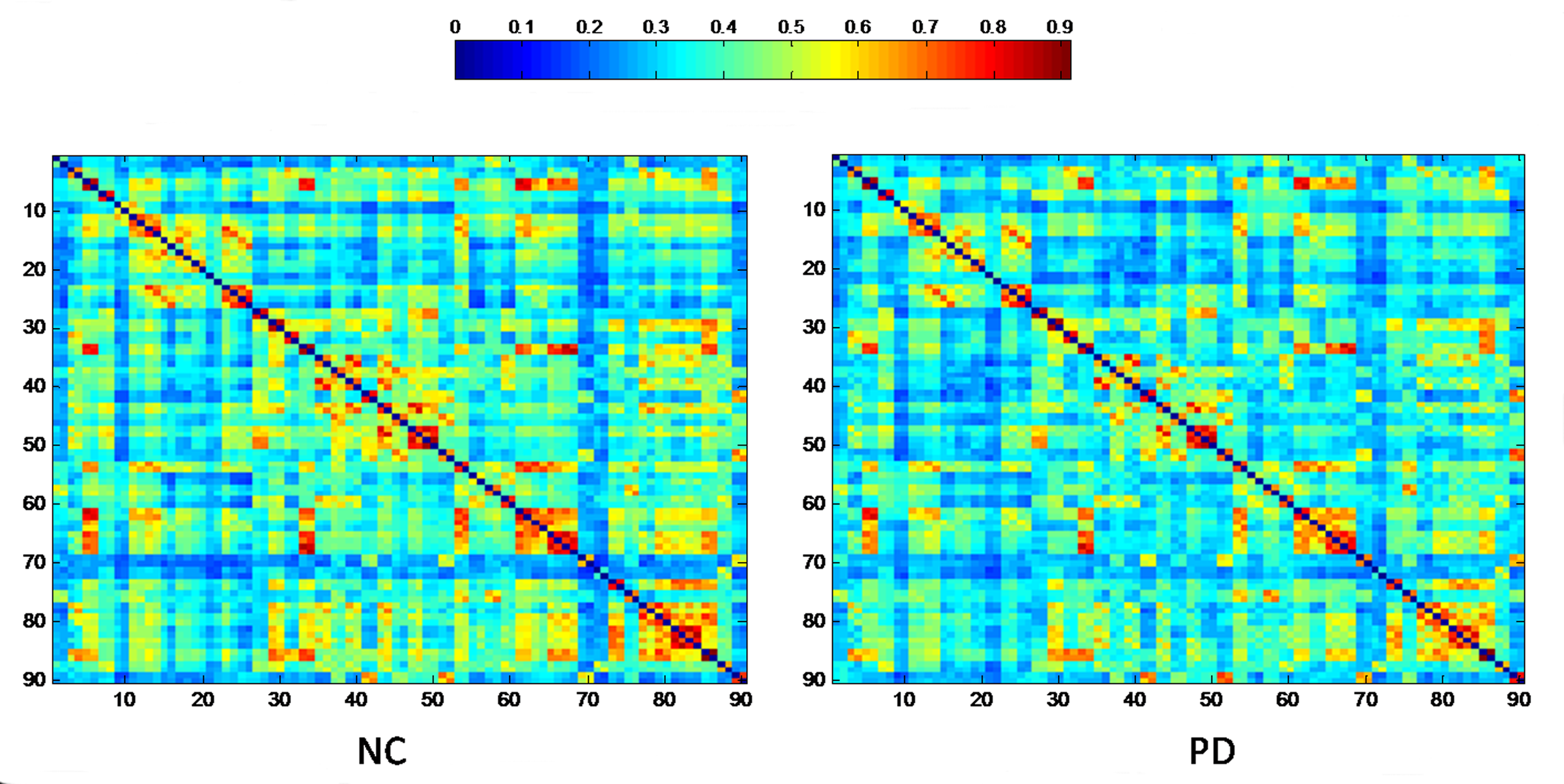

Supplement: S1 Fig — The x and y axes correspond to the ROIs listed in S1 Table. The functional connectivity is indicated with a colorbar. (TIF) [file pone.0141815.s001.tif]
