# Supplementary material for: Alteration of Brain Functional Networks in Early-Stage Parkinson’s Disease: A Resting-State fMRI Study
Source: PLoS One. 2015 Oct 30;10(10):e0141815. doi: 10.1371/journal.pone.0141815 (PMC4627652; doi:10.1371/journal.pone.0141815)
Supplement: S2 Table — (DOCX) [file pone.0141815.s003.docx]

**S2 Table.** Decreased functional connectivity in Parkinson's disease (PD) patients compared with normal controls (p<0.002, uncorrected).

| Region1 | Category | Region2 | Category | t-score |
| --- | --- | --- | --- | --- |
| LING.R | Occipital | HES.L | Temporal | -4.476 |
| SFGdor.L | Frontal | HIP.L | Medial temporal | -3.928 |
| CAL.R | Occipital | THA.L | Subcortical | -3.905 |
| ORBsup.L | Frontal | HIP.L | Medial temporal | -3.877 |
| CAL.R | Occipital | HES.L | Temporal | -3.846 |
| CAL.R | Occipital | THA.R | Subcortical | -3.667 |
| HIP.L | Medial temporal | IPL.R | Parietal | -3.637 |
| ORBsup.R | Frontal | HIP.L | Medial temporal | -3.595 |
| SOG.R | Occipital | HES.L | Temporal | -3.542 |
| PHG.L | Medial temporal | CAL.R | Occipital | -3.51 |
| LING.R | Occipital | THA.R | Subcortical | -3.491 |
| LING.R | Occipital | THA.L | Subcortical | -3.485 |
| HIP.L | Medial temporal | ANG.L | Parietal | -3.462 |
| PUT.L | Subcortical | TPOsup.R | Temporal | -3.439 |
| PUT.L | Subcortical | STG.R | Temporal | -3.429 |
| SFGdor.R | Frontal | CAL.L | Occipital | -3.325 |
| STG.L | Temporal | TPOsup.R | Temporal | -3.299 |
| IPL.L | Parietal | SMG.L | Parietal | -3.283 |
| HIP.R | Medial temporal | ANG.L | Parietal | -3.265 |
| MFG.R | Frontal | OLF.R | Subcortical | -3.262 |
