# Supplementary material for: Alteration of Brain Functional Networks in Early-Stage Parkinson’s Disease: A Resting-State fMRI Study
Source: PLoS One. 2015 Oct 30;10(10):e0141815. doi: 10.1371/journal.pone.0141815 (PMC4627652; doi:10.1371/journal.pone.0141815)
Supplement: S3 Table — (DOCX) [file pone.0141815.s004.docx]

**S3 Table.** The mean values of global efficiency and local efficiency in normal controls (NC) and PD patients groups.

| Sparsity | Global efficiency(NC) | Global efficiency(PD) | E_glob(NC)_/E_glob(PD)_ |
| --- | --- | --- | --- |
| 0.11 | 0.3846 | 0.3672 | 1.047 |
| 0.12 | 0.4054 | 0.3913 | 1.036 |
| 0.13 | 0.4238 | 0.4096 | 1.034 |
| 0.14 | 0.4414 | 0.4272 | 1.03 |
| 0.15 | 0.4597 | 0.4425 | 1.03 |
| 0.16 | 0.4771 | 0.4591 | 1.039 |
| 0.17 | 0.4929 | 0.4744 | 1.038 |
| 0.18 | 0.5065 | 0.4905 | 1.032 |
| 0.19 | 0.5186 | 0.5029 | 1.031 |
| 0.2 | 0.5312 | 0.5139 | 1.033 |
| 0.21 | 0.541 | 0.5253 | 1.029 |
| 0.22 | 0.5521 | 0.5384 | 1.025 |
| 0.23 | 0.5633 | 0.55 | 1.024 |
| 0.24 | 0.5733 | 0.5592 | 1.025 |
| 0.25 | 0.5836 | 0.5697 | 1.024 |
| 0.26 | 0.5928 | 0.5785 | 1.024 |
| 0.27 | 0.6015 | 0.5879 | 1.023 |
| 0.28 | 0.6094 | 0.5965 | 1.021 |
| 0.29 | 0.6188 | 0.6057 | 1.021 |
| 0.3 | 0.6262 | 0.6135 | 1.02 |
| 0.31 | 0.6333 | 0.6212 | 1.019 |
| 0.32 | 0.6407 | 0.629 | 1.018 |
| 0.33 | 0.6475 | 0.6366 | 1.017 |
| 0.34 | 0.6541 | 0.6439 | 1.015 |
| Sparsity | Local efficiency(NC) | Local efficiency(PD) | E_loc(NC)_/E_loc(PD)_ |
| 0.11 | 0.6384 | 0.6343 | 1.006 |
| 0.12 | 0.6582 | 0.6528 | 1.008 |
| 0.13 | 0.6758 | 0.6669 | 1.01 |
| 0.14 | 0.6834 | 0.6814 | 1.002 |
| 0.15 | 0.6968 | 0.6917 | 1.007 |
| 0.16 | 0.7055 | 0.7037 | 1.002 |
| 0.17 | 0.7179 | 0.7123 | 1.007 |
| 0.18 | 0.7296 | 0.7227 | 1.009 |
| 0.19 | 0.7361 | 0.7325 | 1.004 |
| 0.2 | 0.7419 | 0.7394 | 1.003 |
| 0.21 | 0.7505 | 0.7442 | 1.008 |
| 0.22 | 0.7563 | 0.75 | 1.008 |
| 0.23 | 0.7591 | 0.7575 | 1.002 |
| 0.24 | 0.7635 | 0.7641 | 0.999 |
| 0.25 | 0.7681 | 0.7694 | 0.998 |
| 0.26 | 0.7719 | 0.7738 | 0.997 |
| 0.27 | 0.7762 | 0.7784 | 0.997 |
| 0.28 | 0.7782 | 0.7836 | 0.993 |
| 0.29 | 0.7813 | 0.7874 | 0.992 |
| 0.3 | 0.7864 | 0.7918 | 0.993 |
| 0.31 | 0.7898 | 0.7953 | 0.993 |
| 0.32 | 0.7926 | 0.8006 | 0.991 |
| 0.33 | 0.7959 | 0.8046 | 0.989 |
| 0.34 | 0.7993 | 0.809 | 0.988 |
